# The home language environment and early language ability in rural Southwestern China

**DOI:** 10.3389/fpsyg.2022.1010442

**Published:** 2023-03-17

**Authors:** Xinwu Zhang, Yue Ma, Tianli Feng, Vincent Zhang, Xiaoyang Wu, Matthew Li, Queenie Li, Zahra Thani, Lucy Pappas, Sarah-Eve Dill, Scott Rozelle

**Affiliations:** ^1^Stanford Center on China’s Economy and Institutions, Stanford University, Stanford, CA, United States; ^2^School of Management and Economics, University of Electronic Science and Technology of China, Chengdu, Sichuan, China

**Keywords:** home language environment, rural China, child language ability, language environment analysis, early childhood development

## Abstract

Using premier Language Environment Analysis technology to measure and analyze the home language environment, this observational study aims to describe the home language environment and child language ability, drawing on empirical data from 77 households with children aged 18–24 months from rural China. The results show large variation in measures of the home language environment and early language ability, similar to other rural Chinese samples. Results also demonstrate significant correlations between child age and the home language environment, maternal employment and the home language environment, father’s educational attainment and the home language environment, adult–child conversations and early language ability, and child vocalizations and early language ability.

## Introduction

The home language environment, which is comprised of all the language in a given household, is an essential component of early language development. Past research shows that the home language environment is a predictor of early language skills (Weisleder and Fernald, 2013; Ramírez-Esparza et al., 2014; Gilkerson et al., 2018). Specifically, research in Western contexts has demonstrated that children in environments with more adult speech and more adult-child conversations have larger vocabularies and process information faster than their peers (Hart and Risley, 1995; Gilkerson and Richards, 2009; Hurtado et al., 2014). Because early language development has been shown to be significantly correlated to future academic outcomes (Lee and Burkam, 2002; Fernald et al., 2013), the home language environment is an important part of long-term socioeconomic achievement and human development.

Given that the home language environment is key to early language development, differences between unique home language environments might explain variation in language skills between high-and low-socioeconomic status (SES) groups. In the United States, studies have found that low-SES households are consistently characterized by less language input than high-SES families (Hoff, 2003; Rowe and Goldin-Meadow, 2009; Rowe, 2012; Weisleder and Fernald, 2013; Hurtado et al., 2014; Ramírez-Esparza et al., 2014; Hirsh-Pasek et al., 2015; Noble et al., 2015; Ganek and Eriks-Brophy, 2018; Ramírez et al., 2020). Parent–child interactions in higher-SES households also tend to be both more frequent and more engaging (i.e., more questioning or problem-solving conversations posed to children) compared to those in lower-SES households (Saracho, 2017; Romeo et al., 2018, 2021). Due to lower levels of linguistic engagement, children from low-SES families tend to have weaker cognitive and linguistic performance in early childhood compared to children from high-SES backgrounds (Ramey and Ramey, 2004). For example, Fernald et al. (2013) found that 18-24-month old children from low-SES households were trailing behind their peers from high-SES households in language processing efficiency by an average of 6 months. Despite these discrepancies between socioeconomic levels and home language environments, preliminary evidence has found considerable variation in the home language environment of low-SES groups. For example, researchers investigating the home language environments of 29 infants from low-SES families from the United States found large variations in the quantity of overheard speech and child-directed speech (Weisleder and Fernald, 2013). The study also found that despite being from similar socioeconomic backgrounds, some children had stronger language processing skills due to more child-directed speech in their homes (Weisleder and Fernald, 2013).

Studies show that low rates of development are concentrated in areas where there is high exposure to risk factors (infectious disease, malnutrition, poverty) and low availability of high-quality healthcare and educational resources (McCoy et al., 2016; Black et al., 2017; Bai et al., 2019). These areas are typically low-SES settings (McCoy et al., 2016; Black et al., 2017; Bai et al., 2019). Evidence of this can be seen in Black et al. (2017), which found that for children under the age of 5 years, 43% of those living in a developing setting are at risk of ECD delay (Black et al., 2017). Past the first 5 years of life, research shows that adults who were raised in poverty are less likely to be prepared for school and high-skill workforces (Knudsen et al., 2006; Heckman and Masterov, 2007; McCoy et al., 2016; Black et al., 2017; Bai et al., 2019). As research has established that early language skills developed in the first 3 years of life provide a foundation for later skill development and school readiness, we see how the cycle between poverty and ECD delay is perpetuated in low-SES settings (Wake et al., 2012; Meisenberg and Woodley, 2013; Black et al., 2017). Despite limited resources, for low-SES areas, early language skills and the home language environment may be critical economic areas for investment through targeted interventions or developing holistic interventions. For example, the Jamaica Study (Gertler et al., 2014) and the Perry Program (Heckman et al., 2013) significantly improved ECD, education, employment, and economic outcomes in low-SES settings. Moreover, given evidence from rural China that the home language environment is linked to language developmental outcomes (Ma et al., 2021), it may be an important target to positively influence ECD and widespread human development.

While the importance of the home language environment has been made clear by the literature, naturalistic observations are incredibly difficult to capture, and thus quite rare. However, due to the Language ENvironment Analysis (LENA^™^) system, measuring the home language environment has become an easier task for researchers (Gilkerson and Richards, 2009). Using LENA technology, studies from Western and developed settings have identified links between the home language environment and early language development (Gilkerson et al., 2018; Romeo et al., 2018, 2021; d’Apice et al., 2019; Uccelli et al., 2019; Lopez et al., 2020). While LENA has made access to measuring home language environments more accessible, there exists an imbalance between the number of studies conducted in Western and developed settings, and those conducted among less represented non-Western and low-SES samples.

To date, few studies have utilized LENA technology to analyze the home language environment in non-Western samples (Zhang et al., 2015; Pae et al., 2016; Weber et al., 2017; Casillas et al., 2020). Of these non-Western LENA studies, only two have examined populations from China (Zhang et al., 2015; Ma et al., 2021). Zhang et al. (2015) explored the home language environment in Shanghai, and found that after 3 months of intervention, Adult Word Count (AWC) and Conversational Turns Count (CTC) improved. Other findings from this study include significant and positive correlation between CTC and language skills development (measured by Mac-Arthur Bates Communicative Developmental Inventory). The second LENA study investigating the home language environment in China comes from Ma et al. (2021). In this study, the home language environment and early language skills of children aged 20–28 months from low-SES and rural areas of Shaanxi Province were measured, and results indicated significant correlations between the home language environment and child language skills, as well as large variation in LENA measures across households. Overall, the number of studies from China is small in comparison to the number of existing LENA studies, and the lack of numerous studies on rural Chinese populations has emerged as an important gap in the literature that needs to be addressed.

To address this gap, this study analyzes the home language environment and its correlated factors in a rural, non-Western, low-SES environment in rural China. Like many low-SES areas, China faces high rates of developmental delays among children (Yue et al., 2017; Wang et al., 2019). A systematic review and meta-analysis across 14 provinces in rural China found that the risk of language delay was 46% (Emmers et al., 2021). The same study found that developmental delay was strongly associated with low levels of interactive parenting (playing, singing, and reading) from caregivers (Emmers et al., 2021).

Based on past research, the objective of this study is to describe the home language environment and early language abilities of children aged 18–24 months from rural China. To do so, we ask four research questions. First, using LENA audio technology, what is the quality and heterogeneity of the home language environment? Based on past research that suggests the home language environment in rural China is characterized by high variation but lower rates of adult words and conversations than in urban China, we hypothesize that our rural sample will report similar measures to those in rural China, given the similarities in rural backgrounds, cultures, and socioeconomic status (Ma et al., 2021).

Second, we ask what is the distribution of early child language abilities and the prevalence of language delays? A systematic review and meta-analysis of ECD outcomes in rural China indicated that language delay was as high as 46% among children below the age of 5 living in rural households across the entire country (Emmers et al., 2021). We hypothesize that our sample will report rates of delay close to 40%, given these past findings.

Third, what demographic characteristics are correlated to home language environment measures? Research shows that parents often speak more to young girls than boys, given that girls initiate more conversations and make more vocalizations than boys in the first few years of life (Maccoby and Jacklin, 1974; Leaper et al., 1998; Galsworthy et al., 2000; Bornstein et al., 2004; Bleses et al., 2018). Studies also have found significant and positive correlations between child age and child vocalizations, as well as adult–child interactions (Gilkerson et al., 2018; Anderson et al., 2021). For household characteristics, past research has found significant correlations between socioeconomic status factors (i.e., household income, parental education levels, employment status) and measures of the home language environment (Hoff, 2003; Huttenlocher et al., 2010; Weisleder and Fernald, 2013). However, in previous research from rural China, very few significant correlations between household characteristics and the home language environment were identified. The conclusions of Ma et al. (2021) in rural Shaanxi suggested that demographic characteristics could not explain the variability of the home language environment of rural children, and that perhaps the lack of significant associations may be explained by the fact the study focused on a relatively homogeneous population of low-SES families in rural China. Based on such research, we hypothesize that few demographic characteristics will be correlated to the home language environment, but perhaps mother’s age and paternal education level will be correlated to child vocalizations and adult–child conversations (Ma et al., 2021).

Fourth, how does language ability across distinct home language environments differ? Supported by past research from rural China that home language environments with higher rates of adult-child conversations and more child vocalizations are positively correlated to increased rates of language development (Ma et al., 2021), we hypothesize that language ability outcomes will increase given higher rates of conversations and child vocalization.

## Materials and methods

### Sample selection

Data were collected from communities in two rural counties in Southwestern China. The first county is a predominantly rural area, where out of 192,200 local residents, approximately 75% (143,200) have rural *hukou. Hukou*, China’s national household registration system, classifies residents as either rural or urban based on their origins, and determines their residency, property, and social benefits accordingly. In 2019, the annual *per capita* income for residents with rural *hukou* was RMB 19,399 (USD 3,039), which was substantially less than the annual *per capita* income of the first county’s residents with urban *hukou*: RMB 35,707 (USD 5,594) (Hu and Liu, 2020). The second rural county is composed of a predominantly rural population, with a total local population of 1,524,700 people, of which, almost 70% have rural *hukou* (Hu and Liu, 2020). In 2019, the annual *per capita* income for residents with rural *hukou* was RMB 16,413 (USD 2,572), which was less than half the annual *per capita* income for residents in the same area with urban *hukou* (RMB 35,687; USD 5,591) (Hu and Liu, 2020). Compared to annual *per capita* income for rural households in the entire province (RMB 14,670; USD 2,298) and the national average (RMB 16,021; USD 2,510), households from both counties report slightly higher *per capita* incomes (Liu and Ye, 2020). However, these rural households have substantially lower annual *per capita* incomes than urban households in the province (RMB 36,154; USD 5,665) and in the nation (RMB 42,359; USD 6,637) (Liu and Ye, 2020). Regarding educational attainment of the sample, the average amounts of education for people aged 15 and over in the two rural counties are 8.74 and 8.22 years (Meishan Municipal People’s Government, 2020). Both these averages are slightly lower than the averages at the provincial (9.24 years) and the national levels (9.91 years) (National Bureau of Statistics of China (NBS China), 2020). Together, based on income and educational attainment, participants from these counties present a relatively representative sample of low-SES households from rural, Southwestern China.

**Figure 1:**
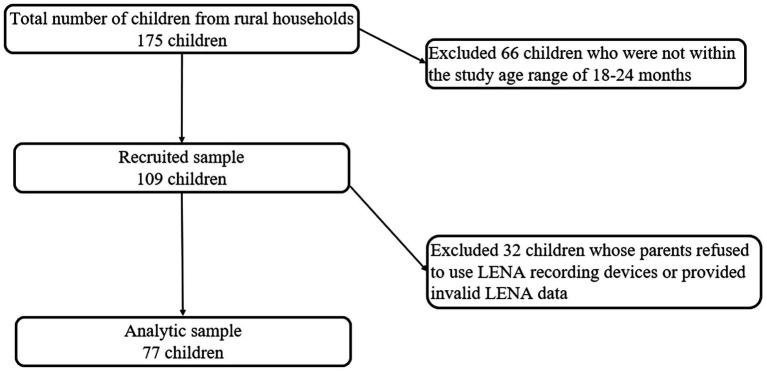
Sampling flowchart for rural children and household selection.

We adopted a three-step strategy for sampling (Figure 1). First, all townships in the two counties were included, except the township in each county that housed the county seat of government which is typically wealthier and more urban than the rest of the county. Second, the research team obtained a list from each township government that listed all households with a child between the ages of 18 and 24 months (Zangl and Fernald, 2007). From this list of 175 households, certain households were excluded from the final analytic sample, including households that did not have rural *hukou*. All remaining eligible children in the age range were enrolled in the study. A total of 109 families were selected through the process described above. Out of this total, 77 families agreed to use LENA technology to participate in the collection of home language environment data. Thus, our final sample included 77 households who participated in both the survey and the assessment of the home language environment. An attrition analysis (see Appendix Table 1) found no significant differences in demographic characteristics between households that participated in the collection of household linguistic environment data (77 households) and those that did not (32 households).

### Data collection

Data were collected in July 2020 by trained survey enumerators who followed a standardized data collection protocol. For each household, the child’s primary caregiver was first identified as the individual most often responsible for the child’s daily care (typically the child’s mother or grandmother). The primary caregiver was administered a quantitative survey that had two main blocks of data: measures of each child’s early language ability and a set of demographic characteristics of the child and household. After the survey was completed, the study team collected data on the home language environment using LENA audio technology, which the caregivers used at home.

To collect the audio data, our research team was extensively trained to follow a standardized data collection protocol and LENA recording process that lasted 4 days. The training support was provided by the LENA Foundation and included but was not limited to teaching the frontline research team how to enter participants (children) with valid licenses, collect good recordings, process recording files, how to set up the LENA Online processing software in China, how to use their ADEX system and how to understand their reports. In addition, our research team had a designated technical support person from the LENA Foundation for the duration of the study who provided technical support throughout the process when needed. On the first day of data collection, researchers interviewed caregivers at the local hospitals or participating caregivers’ homes, using our surveys on language ability and child/household characteristics. After the surveys were completed, our team instructed the families how to use the LENA recording device to record two full days of the home language environment. During the second and third days of surveying, families used the LENA recorders to record the home language environment, charging the recorder overnight as instructed. Finally, on the fourth day, the research team retrieved the LENA recorders and conducted exit interviews with families on their use of LENA recorders.

### Measures

#### The home language environment: LENA

The LENA system makes automatic counts of the following measures: Adult Word Count (AWC), Conversational Turn Count (CTC), and Child Vocalization Count (CVC). AWC calculates the number of adult words spoken near the child, excluding words from electronic devices such as televisions. CTC refers to the number of conversational turns, from one speaker to another, between an adult and the child. CVC measures the number of pre-speech or speech productions made by the child. The recordings could be further broken into segments that include conversations between the focus child and an adult (male or female) using the LENA Advanced-Data Extractor (ADEX; Cunha et al., 2020). Using the LENA ADEX, we generated the number of audio segments initiated by the segment initiator (child, female adult, or male adult) and the number of Conversational Turns (CT) per segment as categorized by who initiated the CT (child, female adult, or male adult). Past literature has demonstrated that LENA recordings are versatile and reliable compared to trained human transcribers in different linguistic contexts, including American English, Spanish, French, Korean, Dutch, and Vietnamese (Xu et al., 2009; Canault et al., 2016; Pae et al., 2016; Busch et al., 2018; Ganek and Eriks-Brophy, 2018; Gilkerson et al., 2018). The accuracy and reliability of LENA software has also been validated and used in Mandarin Chinese (Gilkerson et al., 2015; Zhang et al., 2015; Ma et al., 2021). Results from Gilkerson et al. (2015) found that correlations across LENA and human annotations were strong for AWC (*r* = 0.73) but not as strong for CTC (*r* = 0.22).

For home-based data collection, each child’s primary caregiver was given a special LENA shirt, a fully charged LENA recorder, and a LENA charger. A fully charged LENA recorder can record up to 16 h of continuous audio data, capturing a household’s daily home language environment. In our study, we asked caregivers to record two 16-h days that represented the child’s typical experience at-home. According to the LENA authentication protocol, the recorder is placed in the chest pocket of the specialized shirt that the child wore throughout the day. Caregivers only removed the LENA recorder and specialized LENA shirt while their child bathed or slept at night. After finishing the recording, our investigators retrieved the LENA recorder and interviewed the caregiver to ensure compliance with the LENA recording protocol. All 77 participating families successfully adhered to these protocols, based on post-recording interviews with caregivers and analysis of LENA recordings.

To adjust for skewness common in count data and variations in recording start times among households (Cunha et al., 2020), our study used a four-step process that normalized 16 h of recording to 12 h of audio data. First, we normalized the distribution by using Chebyshev polynomial transformation. Second, using the Least Absolute Shrinkage and Selection Operator (LASSO) regression models, we selected the final Chebyshev polynomials model used in transforming the data. Third, residuals were predicted with the final Chebyshev polynomials model. Finally, we estimated residual count variables from the transformed data and then rescaled them back to the original count metric. Thus, the results of AWC, CTC, and CVC were derived from the adjusted 12-h recordings of each participant. This method is consistent with previous research using the LENA system to study the quality of the home language environment (Gilkerson et al., 2018; Cunha et al., 2020).

#### Early language ability: MCDI

To measure a child’s developing abilities in early language (i.e., vocabulary comprehension, production, gestures, and grammar), we used the Mandarin version of the MacArthur-Bates Communicative Development Inventories (MCDI), a parent-reported assessment which has been adapted and validated in Mandarin (Fenson et al., 2007; Tardif et al., 2008). Caregivers and children from the sampled households spoke Standard Mandarin and/or the provincial dialect of Mandarin. The provincial dialect comes from the Mandarin dialect and shares the same syllable structure as Standard Mandarin, making it widely distinguishable and easily interpreted (Zhang, 2007). Previous studies have used this assessment and demonstrated its reliability in studies of early language development in Chinese children (Zhang et al., 2015; Ma et al., 2021). We utilized the expressive vocabulary assessment of the MCDI for children between 16 and 30 months (our participating children were 18–24 months old). Using a list of 113 words, researchers asked primary caregivers if their children could say each word in Mandarin or the provincial dialect; each word the child could say counted for one point. By comparing the parent-reported results with empirically determined cutoff scores established using the MCDI manual (Tardif et al., 2008), the status of a child’s expressive language developmental progress (rate of delay) was determined. Rates of delay were established for each age group using group cutoff scores from Tardif et al. (2008) (any child under the 10th percentile of language development in their one-month age group was considered delayed) and then combined to produce a full sample cutoff for delay.

#### Demographic information

For each child, we recorded their age in months, gender, and prematurity status. The demographic information survey also collected data on household characteristics, including the mother’s age (in years), the mother’s highest level of educational attainment, whether the mother was employed at the time of surveying, whether the mother was the primary caregiver, the father’s highest level of educational attainment, whether the father had lived at home for at least 6 months of the last year, the total number of adults in the household, and the household asset index score. The household asset index was based on whether the family owned or had access to running water, a toilet, a water heater, a washing machine, a computer, internet access, a refrigerator, air conditioning, a motorcycle, and a car/truck. The final household asset index was generated using polychoric principal component analysis (PCA) (Kolenikov and Angeles, 2009).

Our focus on these demographic characteristics is rooted in the literature. Child age and gender were collected because previous studies have found differences in the language development outcomes between older and younger children (Gilkerson et al., 2018) and between girls and boys (Zhang et al., 2015; Yue et al., 2017). Parental characteristics including age, educational attainment, and migration status have also been shown to be associated with child language development (Lee and Burkam, 2002; Weisleder and Fernald, 2013; Ramírez et al., 2020). SES has been shown to be associated with early childhood development as well (Yue et al., 2017; Gilkerson et al., 2018). Finally, the number of adults in the household was collected as previous studies suggest that household size is an influential factor in the home language environment and language development (McCartney, 1984; Mayor et al., 2018). To determine whether the inclusion of these demographic information variables was valid for our study, we used Kernel density plots to test the distribution of the continuous covariates and have analyzed the variances of the binary covariates. We found that all continuous covariates are normally distributed, and that the variance of all binary covariates are appropriate for use in our analysis.

### Statistical analysis

There are four parts to our statistical analysis. First, to describe the quality and heterogeneity of the home language environment, we graphically present the AWC, CTC, and CVC scores of participating families in rank order. Second, to examine the nature of early language ability and prevalence of language delay we graphically demonstrate the distribution of children’s MCDI scores. For both the LENA and MCDI distributions, we describe the standard deviations (SDs), means, and ranges of each key variable. Third, to analyze demographic characteristics associated with different home language environments, we conducted *t*-tests to compare the demographic characteristics of children and families in the top and bottom quartiles of AWC, CTC, and CVC. Lastly, we analyzed the correlations between home language environment and early language ability using an additional *t*-test that contrasted the MCDI scores of children in the upper and lower terciles of AWC, CTC, and CVC. We utilized STATA 16.1 to perform all statistical analyses. *p*-values at or below 0.05 were statistically significant.

## Results

### Descriptive statistics of children and households

Table 1 displays the descriptive statistics of the 77 sample children and households. The children in the study were on average 22 months old (SD = 1.6). About 60% of the children in the study were male, and 13% were born prematurely. In regard to mothers, their average age was 28 years (SD = 4.3), and 61% had jobs at the time of the survey. The mothers were the primary caregivers of the child in 48% of the sample households. About 51% of mothers completed high school or above, compared to 47% of fathers. In 31% of households, the father had lived at home for at least 6 months of the previous year. The average number of adults in each household was two (SD = 1.1).

**Table 1:** Descriptive statistics of household characteristics (*N* = 77).

Variables	*N*/mean	Percent/SD
	(1)	(2)
***Child characteristics***		
Age (months)	21.88	1.60
Gender		
Male	46	59.74
Female	31	40.26
Prematurity		
Yes	10	12.99
No	67	87.01
***Household characteristics***		
Age of mother (years)	27.52	4.25
Maternal education		
Middle school or below	38	49.35
High school or above	39	50.65
Mother has a job		
Yes	47	61.04
No	30	38.96
Mother is the primary caregiver		
Yes	37	48.05
No	40	51.95
Paternal education		
Middle school or below	41	53.25
High school or above	36	46.75
Father lived at home for at least 6 months of the past year		
Yes	24	31.17
No	53	68.83
Number of adults in the household	2.43	1.08
Asset index (PCA score)	0.00	1.37

### The home language environment

**Figure 2:**
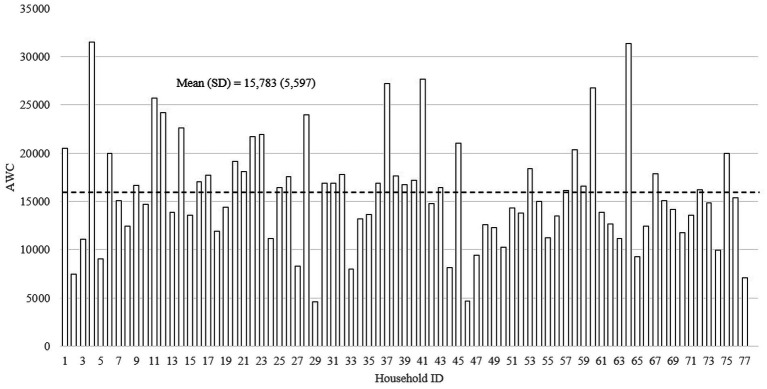
The distribution of Adult Word Count (*N* = 77).

**Figure 3:**
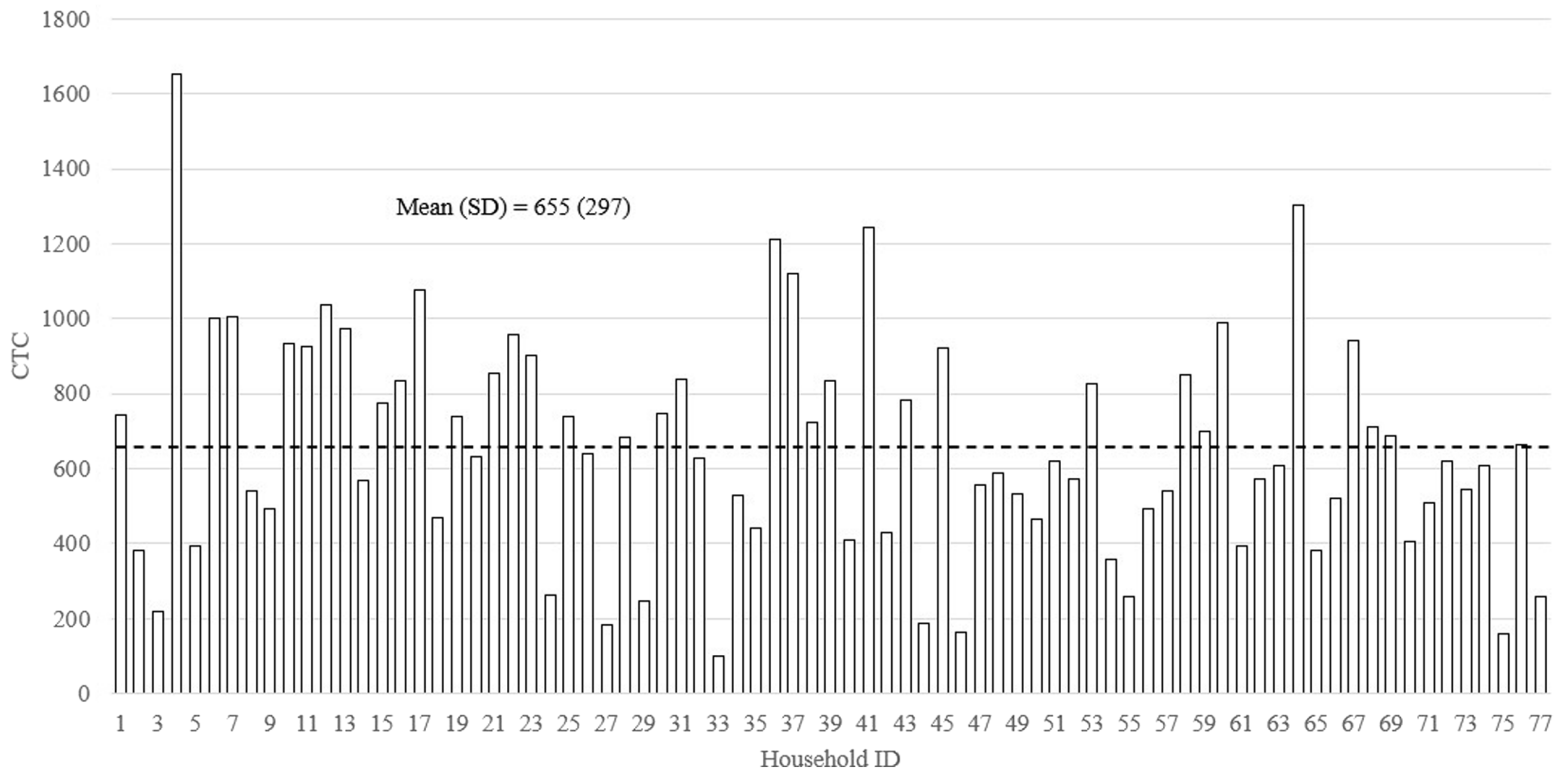
The distribution of Conversational Turn Count (*N* = 77).

**Figure 4:**
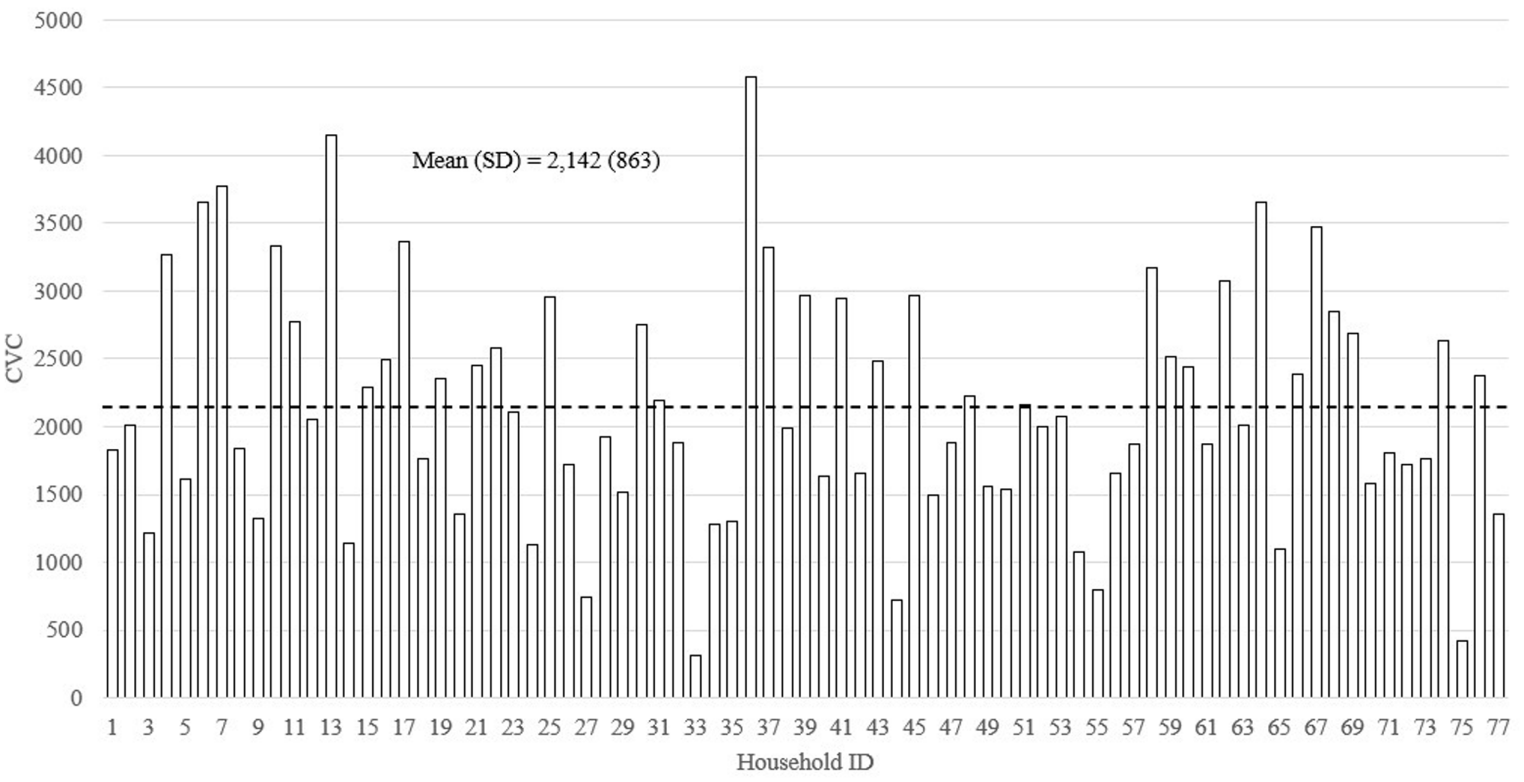
The distribution of Child Vocalization Count (*N* = 77).

Figures 2–4 show the distributions of the three home language environment metrics among the sampled households—AWC, CTC, and CVC, respectively. As shown in Figure 2, the mean AWC of the sample was 15,783 words (SD = 5,597), and there was a 2.1-fold difference between the top tercile (AWC = 21,737) and bottom tercile (AWC = 10,152) participants. The difference in AWC was significant at *p* < 0.001. Reported in Figure 3, the average CTC was 655 (SD = 297) with a 2.9-fold difference between the top tercile (CTC = 982) and the bottom tercile (CTC = 344). The difference between the highest and the lowest CTC score was significant at *p* < 0.001. Finally, Figure 4 displays the mean score for CVC was 2,142 (SD = 863), and the difference in CVC between the top tercile (CVC = 3,111) and bottom tercile (CVC = 1,251) scores was 2.5-fold. Similar to the difference in CTC, the difference in CVC was significant at *p* < 0.001.

Appendix Table 2 presents the summary statistics and percentiles for LENA-generated initiation of conversations in audio segments. Columns 3 through 7 present the means for the 5th, 25th, 50th, 75th and 95th percentiles of the sample, respectively. The results show significant variation in the number of CTs and audio segments across the sample. For the number of CTs per segment as categorized by the child, a female adult, or a male adult, the average child-initiated CT among the 95th percentile (599) was 7 times larger than the 5th percentile (76); the average female-initiated CT in the 95th percentile (485) was 7 times larger than the 5th percentile (66); and the average male-initiated CT of the 95th percentile (194) was more than 27 times larger than the 5th percentile (7). In addition, the average child-initiated CT across all percentiles was similar to the female-initiated CT, while across all percentiles the female-initiated CT was greater than male-initiated CT.

For the number of audio segments initiated by the segment initiator (the child, female adult, or male adult), the average child-initiated segment in the 95th percentile (733) was 3 times larger than in the 5th percentile (189); the average female-initiated segment in the 95th percentile (588) was 3 times larger than the 5th percentile (182); and the average male-initiated segment of the 95th percentile (262) was more than 14 times larger than the 5th percentile (18). Similarly, the average number of child-initiated segment of all percentiles was close to female-initiated segments, however the number of female-initiated segments was greater than the number of male-initiated segments.

### Early language ability

**Figure 5:**
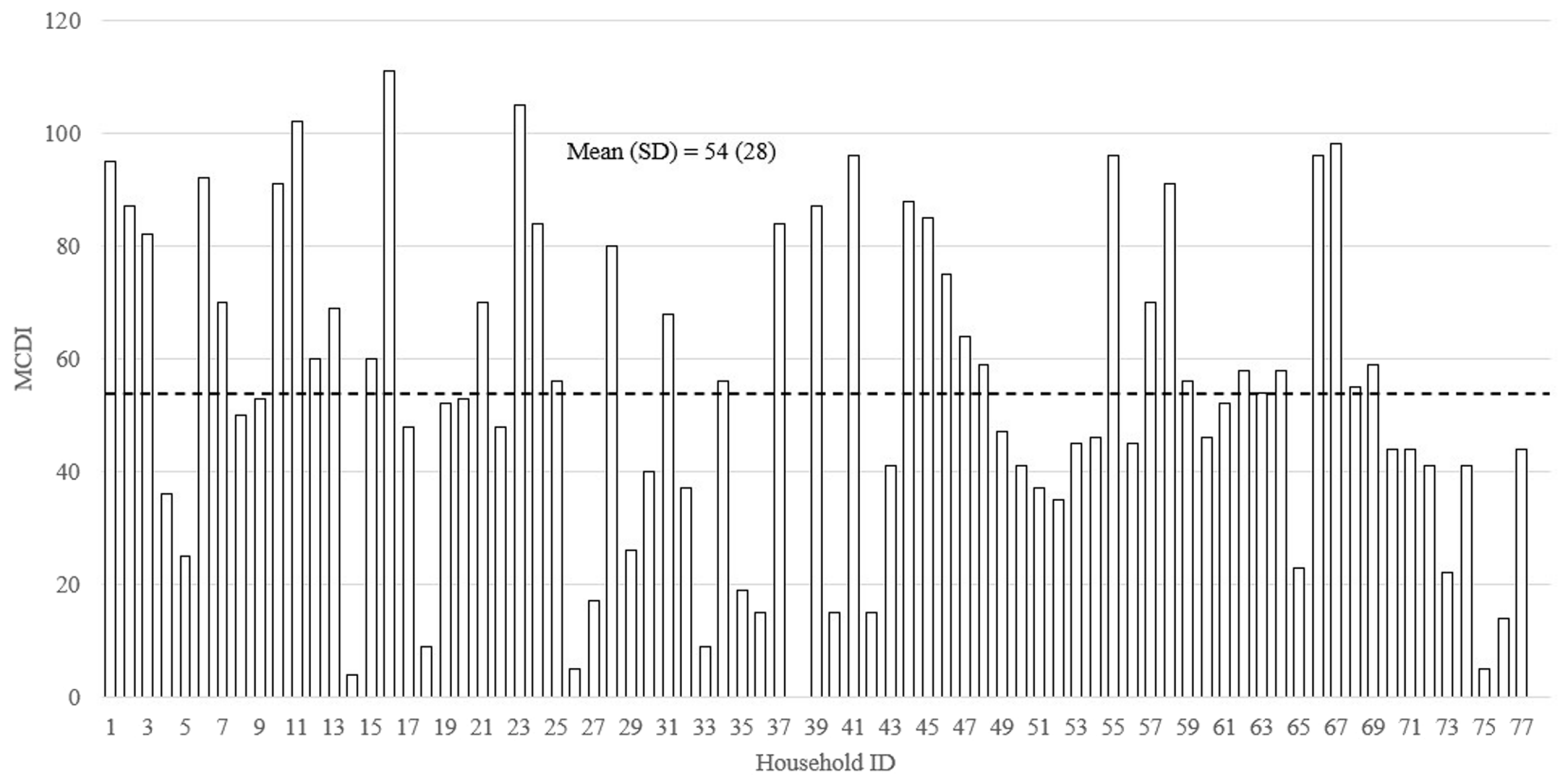
The distribution of MacArthur-Bates Communicative Development Inventories scores (*N* = 77).

Figure 5 presents the MCDI score distribution of the sample children. The mean MCDI score was 54 (SD = 28). The status of a child’s early language ability was obtained by comparing the parent-reported results with the empirically determined cutoff score established by Tardif et al. (2008). About 19.5% of children were below the CDI cutoff for proficient language development (Tardif et al., 2008). There was also a 3.7-fold (p < 0.001) difference between the bottom tercile (MCDI = 23) and top tercile (MCDI = 86), revealing high MCDI score variation in the sample.

### Correlates of variation in the home language environment

Tables 2–4 present the results of our descriptive *t*-tests comparing demographic characteristics of sample households in the top and bottom terciles of AWC, CTC, and CVC, respectively. Table 2 compares households in the top and bottom terciles of AWC. The results show that the only significant variable of interest was father education. Children of fathers who had completed high school were significantly more likely to have AWC scores in the top tercile than those whose fathers did not (*p* < 0.01); specifically, children were 38% more likely to be in the top tercile of AWC if their fathers had completed high school. The results of our descriptive *t*-test comparing the demographic characteristics of households in the top and bottom terciles of CTC are shown in Table 3. The child’s age was the only statistically significant variable among CTC scores. While the top tercile consisted largely of children that were 22 months old, the bottom tercile consist largely of children that were 21 months old (*p* < 0.05). Table 4 compares the characteristics of sample children and households in the top and bottom terciles of CVC. Similar to the results regarding CTC, children who were older were significantly more likely to be in the top tercile of CVC (*p* < 0.01) than younger children. The average age for the top tercile of CVC scores was 22.5 while the average age for the bottom tercile was 21.2, revealing a difference of 1.2 months between the two groups.

**Table 2:** Differences in demographic characteristics between top and bottom terciles of Adult Word Count (AWC).

Variables	Bottom tercile AWC	Top tercile AWC	Difference
	(1)	(2)	(3) = (2)–(1)
**Child characteristics**			
Age (months)	21.671 [1.472]	22.197 [1.686]	0.527 (0.450)
Gender (1 = boy)	0.400 [0.500]	0.654 [0.485]	0.254 (0.134)
Prematurity (1 = yes)	0.080 [0.277]	0.154 [0.368]	0.074 (0.096)
**Household characteristics**			
Age of mother (years)	27.400 [3.731]	28.077 [5.091]	0.677 (1.200)
Maternal education (1 = completed high school or above)	0.520 [0.510]	0.423 [0.504]	−0.097 (0.142)
Mother has a job (1 = yes)	0.520 [0.510]	0.692 [0.471]	0.172 (0.138)
Mother is the primary caregiver (1 = yes)	0.480 [0.510]	0.423 [0.504]	−0.057 (0.142)
			
Paternal education (1 = completed high school or above)	0.240 [0.436]	0.615 [0.496]	0.375^**^ (0.135)
Father lived at home for at least 6 months of the past year (1 = yes)	0.360 [0.490]	0.346 [0.485]	−0.014 (0.131)
Number of adults in the household	2.56 [0.961]	2.308 [1.320]	−0.252 (0.306)
Asset index (PCA score)	0.258 [0.922]	−0.298 [1.795]	−0.556 (0.383)

** means that the coefficient is significant at 1%.

* means that the coefficient is significant at 5%.

Standard deviations in the brackets. Standard errors in parentheses.

**Table 3:** Differences in demographic characteristics between top and bottom terciles of Conversational Turn Count (CTC).

Variables	Bottom tercile CTC	Top tercile CTC	Difference
	(1)	(2)	(3) = (2)–(1)
**Child characteristics**			
Age (months)	21.441 [1.695]	22.472 [1.563]	1.030^*^ (0.438)
			
Gender (1 = boy)	0.600 [0.500]	0.769 [0.430]	0.169 (0.134)
			
Prematurity (1 = yes)	0.160 [0.374]	0.077 [0.272]	−0.083 (0.095)
**Household characteristics**			
Age of mother (years)	26.920 [4.163]	27.808 [4.060]	0.888 (1.200)
Maternal education (1 = completed high school or above)	0.520 [0.510]	0.462 [0.508]	−0.058 (0.143)
Mother has a job (1 = yes)	0.520 [0.510]	0.731 [0.452]	0.211 (0.137)
Mother is the primary caregiver (1 = yes)	0.480 [0.510]	0.423 [0.504]	−0.057 (0.142)
Paternal education (1 = completed high school or above)	0.320 [0.476]	0.577 [0.504]	0.257 (0.139)
Father lived at home for at least 6 months of the past year (1 = yes)	0.360 [0.490]	0.308 [0.471]	−0.052 (0.132)
Number of adults in the household	2.520 [0.963]	2.385 [1.267]	−0.135 (0.306)
Asset index (PCA score)	−0.031 [1.318]	0.079 [1.544]	0.110 (0.388)

** means that the coefficient is significant at 1%.

* means that the coefficient is significant at 5%.

Standard deviations in the brackets. Standard errors in parentheses.

**Table 4:** Differences in demographic characteristics between top and bottom terciles of Child Vocalization Count (CVC).

Variables	Bottom tercile CVC	Top tercile CVC	Difference
	(1)	(2)	(3) = (2)–(1)
**Child characteristics**			
Age (months)	21.232 [1.649]	22.505 [1.348]	1.273^**^ (0.430)
Gender (1 = boy)	0.640 [0.490]	0.769 [0.430]	0.129 (0.132)
Prematurity (1 = yes)	0.080 [0.277]	0.077 [0.272]	−0.003 (0.094)
**Household characteristics**			
Age of mother (years)	27.200 [4.646]	28.231 [3.314]	1.031 (1.197)
Maternal education (1 = completed high school or above)	0.520 [0.510]	0.538 [0.508]	0.018 (0.143)
Mother has a job (1 = yes)	0.480 [0.510]	0.731	0.251
		[0.452]	(0.136)
Mother is the primary caregiver (1 = yes)	0.520 [0.510]	0.385 [0.496]	−0.135 (0.141)
Paternal education (1 = completed high school or above)	0.320 [0.476]	0.538 [0.508]	0.218 (0.140)
Father lived at for at least 6 months of the past year (1 = yes)	0.440 [0.507]	0.346 [0.485]	−0.094 (0.128)
Number of adults in the household	2.480 [0.918]	2.654 [1.231]	0.174 (0.301)
Asset index (PCA score)	0.077 [1.439]	−0.072 [1.389]	−0.149 (0.388)

** means that the coefficient is significant at 1%.

* means that the coefficient is significant at 5%.

Standard deviations in the brackets. Standard errors in parentheses.

Table 5 illustrates results of multivariate correlations between household characteristics and the home language environment. Results show that child’s age was not statistically significantly correlated with AWC but was statistically significantly correlated with CTC and CVC at *p* < 0.05 and *p* < 0.01, respectively. The table also shows that paternal education was statistically significantly correlated with AWC and CTC. Additionally, we also found a significant positive correlation between maternal job status and AWC (*p* < 0.05).

**Table 5:** Multivariate correlations between household characteristics and Adult Word Count (AWC), Conversational Turns Count (CTC), and Child Vocalization Count (CVC).

Variables	AWC	CTC	CVC
	(1)	(2)	(3)
**Child characteristics**			
Age (months)	698.750	52.105^*^	190.111^**^
	(393.421)	(20.856)	(62.524)
Gender (1 = boy)	2,393.342	125.485	391.994
	(1,339.005)	(70.984)	(212.800)
Prematurity (1 = yes)	−104.596	−151.146	−355.836
	(1,879.177)	(99.621)	(298.646)
**Household characteristics**			
Age of mother (years)	63.214	2.390	1.872
	(160.084)	(8.487)	(25.441)
Maternal education (1 = completed high school or above)	−820.719	−42.208	−54.100
	(1,362.850)	(72.249)	(216.589)
Mother has a job (1 = yes)	4,656.466^*^	136.359	181.077
	(2,225.055)	(117.956)	(353.614)
Mother is the primary caregiver (1 = yes)	3,024.422	−2.393	−148.629
	(2,243.781)	(118.949)	(356.590)
Paternal education (1 = completed high school or above)	2,956.582^*^	164.855^*^	310.129
	(1,254.894)	(66.525)	(199.432)
Father lived at home for at least 6 months of the past year (1 = yes)	624.804	53.320	72.584
	(1,616.531)	(85.697)	(256.905)
Number of adults in the household	−693.142	−43.026	26.188
	(678.162)	(35.951)	(107.776)
Asset index (PCA score)	−621.744	−0.250	−31.342
	(521.417)	(27.642)	(82.866)
Observations	77	77	77
R-square	0.241	0.245	0.195

** means that the coefficient is significant at 1%.

* means that the coefficient is significant at 5%.

Standard errors in parentheses.

### The home language environment and early language ability

**Figure 6:**
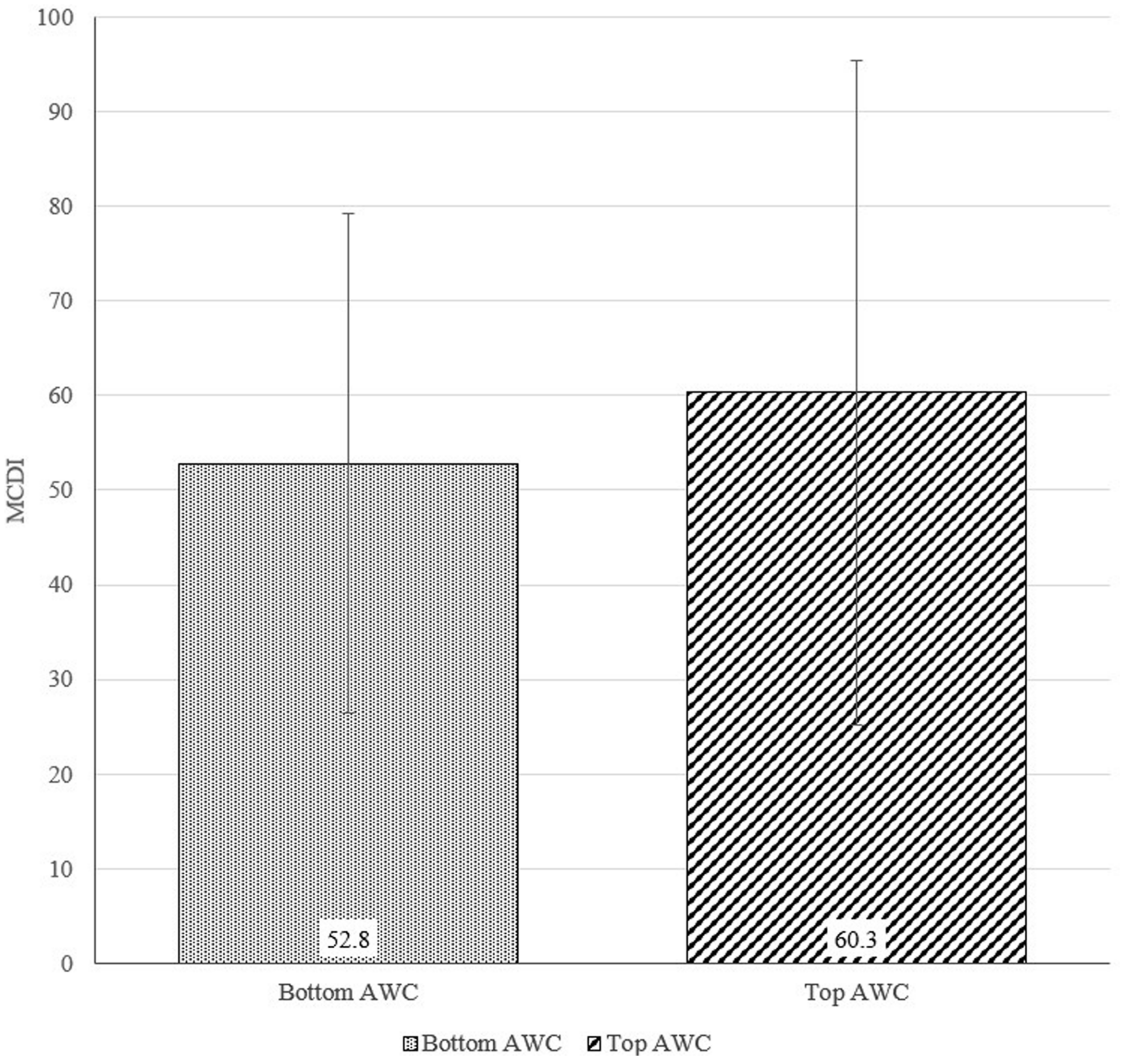
Differences in MacArthur-Bates Communicative Development Inventories (MCDI) scores between top and bottom terciles of Adult Word Count (AWC; *p*-value = 0.391).

**Figure 7:**
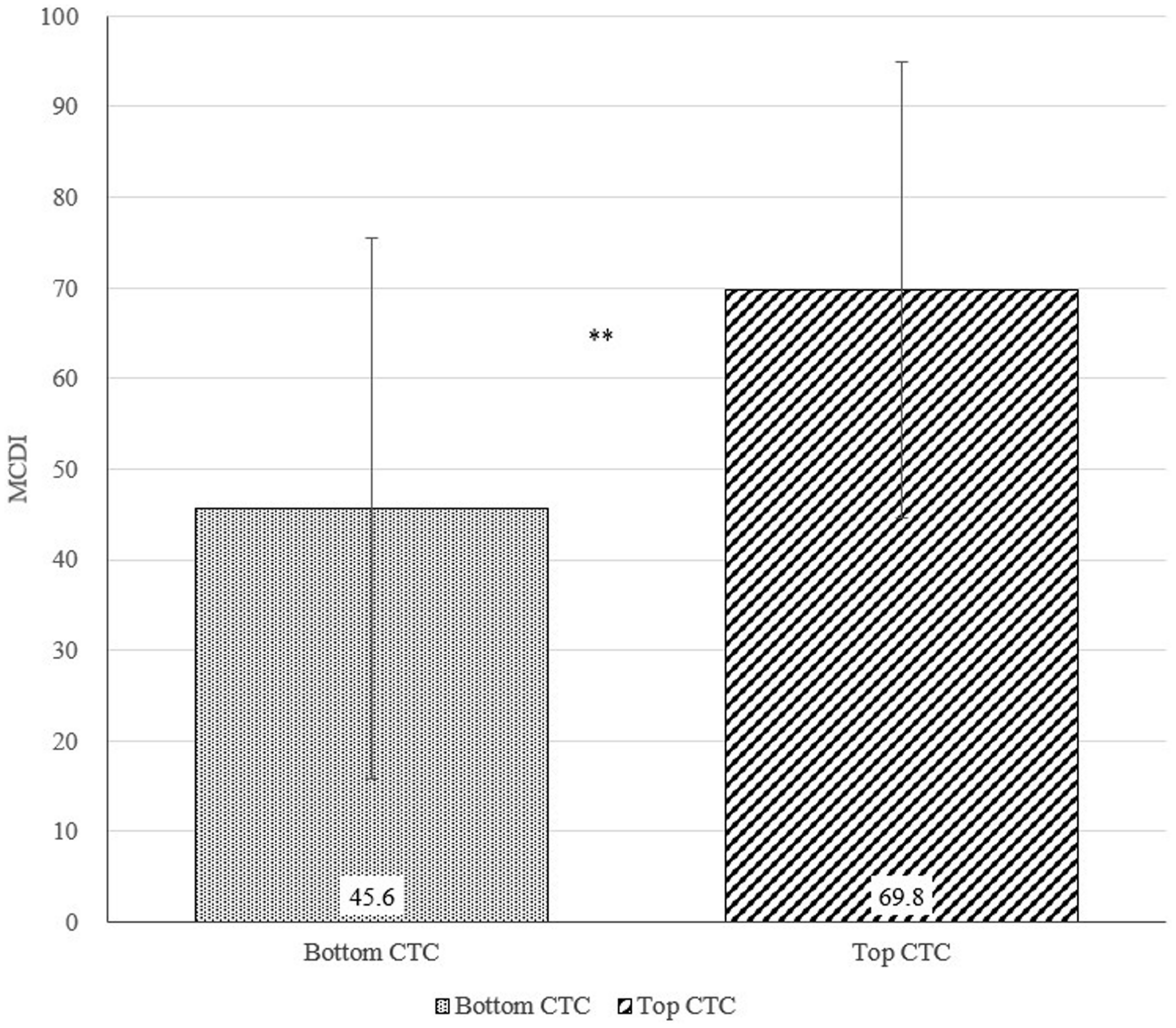
Differences in MacArthur-Bates Communicative Development Inventories (MCDI) scores between top and bottom terciles of Conversational Turn Count (CTC; *p*-value = 0.003).

**Figure 8:**
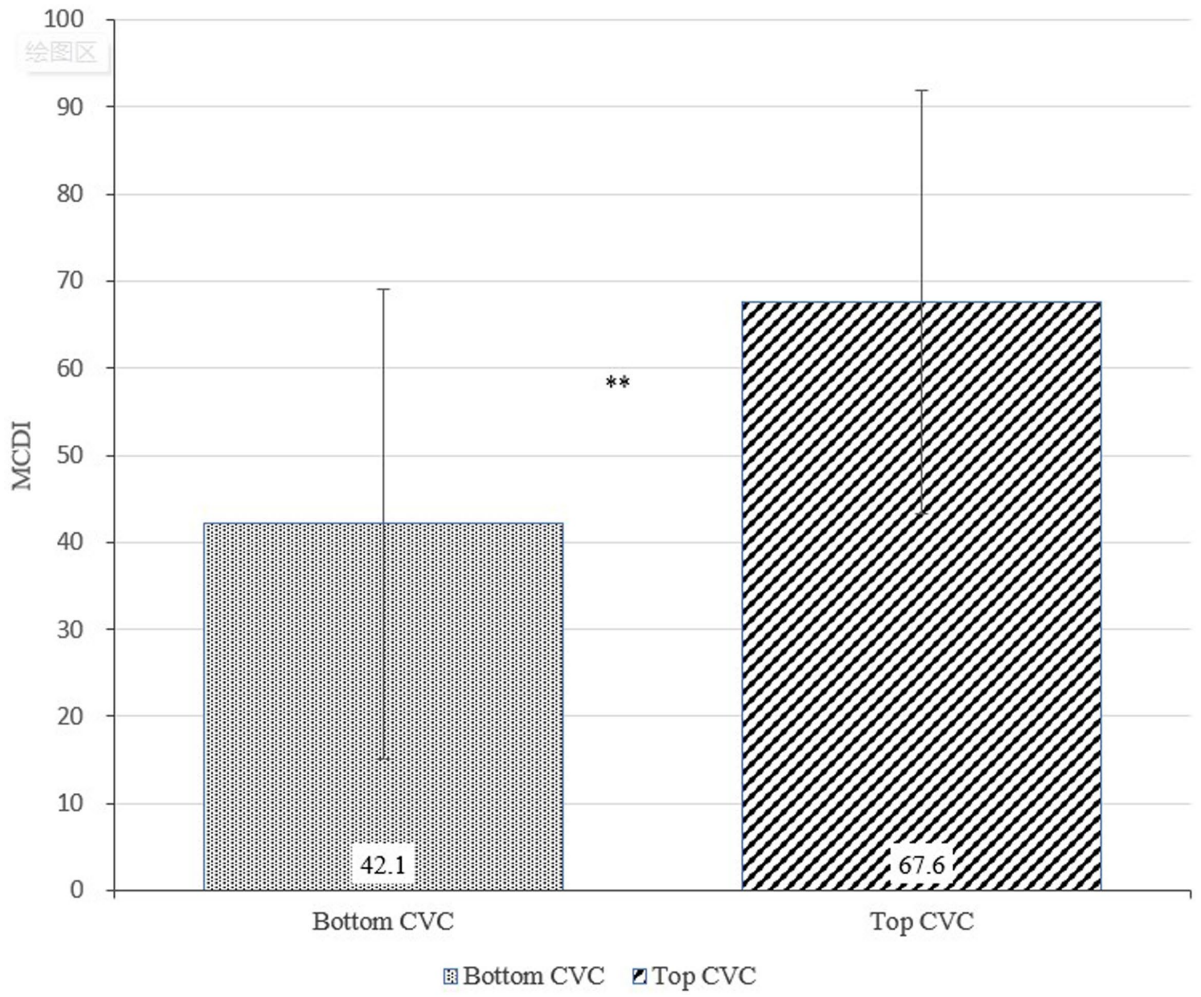
Differences in MacArthur-Bates Communicative Development Inventories (MCDI) scores between top and bottom terciles of Child Vocalization Count (CVC; *p*-value = 0.001).

Figures 6–8 show the results of the *t*-tests comparing early language ability between the top and bottom terciles of AWC, CTC, and CVC, respectively. Children in the top tercile of AWC had higher MCDI scores than children in the bottom tercile of AWC (Figure 6). However, AWC was not significantly correlated with MCDI (*p* = 0.391). Figure 7 shows the difference in MCDI scores between top and bottom terciles of CTC. The difference is 24.2 as children in the top tercile of CTC had an average MCDI score of 69.8 and those in the bottom tercile of CTC had an average MCDI score of 45.6. Finally, for CVC (Figure 8), the participants in the top tercile had an average MCDI score of 67.6 compared to an average MCDI score of 42.1 for participants in the bottom tercile; thus, the difference of MCDI top and bottom tercile scores is 25.5. In other words, the differences between top and bottom terciles for CTC and CVC were statistically significant (*p* = 0.003 for CTC and *p* = 0.001 for CVC). Furthermore, holding control variables constant, we found a strong association between CTC and CVC, and MCDI. Appendix Table 3 illustrates that AWC was not statistically significant while CTC and CVC were statistically significance at *p* < 0.05. Overall, these findings show a positive correlation between certain elements of the home language environment and early language ability.

## Discussion

This observational study of 77 children aged 18–24 months reveals findings on variations in the home language environment and early language abilities children living in low-SES households in a non-Western, rural setting. To reiterate the main objectives of our study, we first measured the heterogeneity of the home language environment in our sample households using LENA equipment. Second, we examined the language ability of the sample children and the prevalence of language delay in the sample. Third, we compared the demographic characteristics of households in the top and bottom terciles of the home language environment measures. Last, we compared the language ability of children across households with home language environments in the aforementioned terciles. Overall, the rural sample’s home language environment was most significantly correlated to maternal employment, father educational attainment, and child age. The sample households had large and substantial variation across all measures of home language environment, despite having similar demographic characteristics (i.e., income level, educational level). The results show a strong positive association between CTC and CVC, and early language ability.

Despite having relatively similar household characteristics, such as low levels of parental educational attainment and living in low-SES neighborhoods, there was large and substantial variation across all measures of home language environments households. Between the highest and lowest ranking child for each home language environment measure, the sample had a 6.8-fold difference in AWC, a 16.5-fold difference in CTC, and a 14.6-fold difference in CVC. The substantial variation found in this study is consistent with other studies examining the home language environment of low-SES communities (Pan et al., 2005; Hurtado et al., 2008; Weisleder and Fernald, 2013; Ma et al., 2021); however, the variations were far greater than those found when studying a low-SES sample from another rural community in China. The variation identified in the home language environments from rural Shaanxi in Ma et al. (2021) was a 5-fold difference between the highest and lowest ranking child in both the AWC and CVC measures, and 6.6-fold difference in the CTC measure. All of these differences are smaller than those identified in our results. However, analyzing the home language environments of 29 low-SES Spanish-learning children in the U.S., Weisleder and Fernald (2013) discovered a 15-fold difference between the highest and lowest ranking child in terms of AWC—a variation greater than that of this study sample. In other words, substantial and heterogeneous home language environment results within similarly disadvantaged, low-SES groups are common across diverse linguistic, cultural, and geographic contexts, and confirms our findings.

In addition to finding high variation in the home language environment, evidence from this study indicates that measures of the home language environment in rural households were somewhat higher than those of previously studied households in low-SES rural China, but ultimately representative of lower-SES rural households. In a sample containing 38 children aged 20–27 months old from rural Shaanxi Province in Northwestern China, Ma et al. (2021) reported that the children heard 14,739 adult words, engaged in 611 conversational turns, and generated 2,332 vocalizations throughout 16-h recordings, on average. In comparison, our sample’s rural households showed slightly greater results: children heard 15,783 adult words, engaged in 655 conversational turns during the same time duration. Our sample did not show higher counts of child vocalizations, however, as children in our sample only produced 2,142 vocalizations. While there is slight variation between measures of the home language environment between these two samples, the counts are similar, and together give a clearer picture of the home language environment in rural Western China.

Furthermore, this study found overall low levels of expressive vocabulary ability but high levels of variation as measured by MCDI. Less than 20% (19.5%) of the sampled children were below the MCDI cutoff for proficient language development (Tardif et al., 2008). Moreover, the sample had high heterogeneity, with a 3.7-fold difference between participants scoring in the first and third quartiles. On average, participants in the first quartile had a MCDI score of 23, while third quartile participants scored at 86. Our observed variation is larger than the variation seen in the poor rural China sample from Ma et al. (2021), which reported an approximately 2-fold difference between the first (MCDI = 28) and third quartiles (MCDI = 55). Thus, this further indicates the heterogeneity of children’s early language abilities present in low-SES communities. Given the links between the home language environment and early language abilities of young children (Weisleder and Fernald, 2013; Ramírez-Esparza et al., 2014; Gilkerson et al., 2018), our data suggests that low-SES groups living in rural China, where the home language environment is characterized by low rates of conversation and child vocalization, might be at heightened risk of language delay.

Ultimately, our findings indicate that of all the demographic characteristics, child age, mother’s employment status, and the level of paternal education have significant correlations with the home language environment. On average, older children have more conversations with adults, and make more vocalizations. Other research has similarly found that older children tend to have increased language abilities (Gilkerson et al., 2018; Anderson et al., 2021). Next, maternal employment was significantly correlated to AWC. In the literature, maternal employment has been correlated to higher levels parental self-efficacy (Jackson and Scheines, 2005), which is positively associated with parent–child interactions, including verbal interactions (Des Jardin and Eisenberg, 2007). Despite their working hours, employed mothers may talk to their child more frequently and interactively because they feel more confident in their abilities to parent, thus providing higher levels of investment in their child’s home language environment. While this is one possible reason for the relation between a mother’s employment and the home language environment, relation between these two factors has yet to be directly studied, especially in samples like ours. Thus, we only provide preliminary hypotheses for what may underlie the correlation. Examining paternal education, we found it to be a positive and statistically significant correlate of AWC. Little research has focused on paternal speech and its effect on early language abilities, however, studies have found that parent education does significantly and positively correlate to the quantity of words spoken to children, such that the more years of schooling correlates to higher counts of words spoken (Hoff, 2003; Huttenlocher et al., 2010; Rowe, 2012).

An interesting finding of this study is the overall lack of significant correlations between household and child characteristics and measures of the home language environment. Out of 11 possible covariates rooted in the literature as being significant for the home language environment or early language development, only three variables were found to be significant. Past research from rural China can confirm a lack of significant demographic characteristics for home language environment measures. Ma et al. (2021) reported similarly few significant correlations between demographic characteristics and the home language environment, suggesting that perhaps due to the homogeneity of a low-SES, rural sample in Shaanxi Province, there were no significant covariates able to be identified. Perhaps that is the same situation for our sample. Another factor could be the influence of non-interactive parenting practices that is common among rural Chinese households. A systematic review and meta-analysis of ECD studies across rural China identified significantly low levels of stimulating parenting practices for children below the age of 5 years in rural China (Emmers et al., 2021). This meta-analysis, Emmers et al. (2021), found that parents rarely read books, told stories, or sang songs to their children, all of which would influence the home language environment and have been identified in the literature as significant parenting practices for language development outcomes (Bradley et al., 2011; Grantham-McGregor and Smith, 2016; Emmers et al., 2021). Therefore, future research in a larger sample size in rural China may illuminate potential correlations between demographic characteristics and the home language environment.

Similar to previous studies (Hart and Risley, 1995; Greenwood et al., 2011; Weisleder and Fernald, 2013; Ma et al., 2021), our research found strong positive correlations between the home language environment and early language ability. The differences of MCDI scores across CTC and CVC between the top tercile and the bottom tercile participants are both statistically significant (*p* < 0.01). For AWC, CTC, and CVC, children in the top tercile scored higher than children in the bottom tercile, with 7.5, 24.2, and 25.5 MCDI points higher, respectively. Demonstrating the importance of caregiver–child interaction and child-directed speech in early childhood development, these results stress the critical finding that conversations and subsequent child vocalizations, may be more significantly important for early language ability than the sheer quantity of words heard by children. Recent literature on the home language environment supports this finding (Romeo et al., 2018; Ramírez et al., 2020; Gómez and Strasser, 2021), as conversations have emerged as the most critical aspect of language environments for a child’s developmental trajectory.

### Strengths and limitations

We acknowledge several strengths of this study. First, this research is one of the first studies to quantitatively measure the home language environment with LENA technology in a non-Western, low-SES setting in China. Second, and importantly, this study quantitatively examines the home language environment and early language abilities of children from rural China, one of the largest and most developmentally vulnerable populations in China. Given China’s vast socioeconomic gaps between rural and urban populations, this study details how children from low-SES communities are disadvantaged in early childhood (Xie and Zhou, 2014) and provides greater context on the early language ability of this population.

This research also has several limitations. The first limitation is the duration of LENA recordings. Using LENA recorders, we recorded only 2 days of audio for each sample household, compared to the weekly or biweekly recording of other studies (Zhang et al., 2015; Suskind et al., 2016). However, before recording, we asked all caregivers whether the next 2 days would be representative of their daily home life. If not, the caregivers would wait until they had 2 days of stable, daily life so that the LENA recorders could capture sample children’s most typical at-home experiences. Additionally, our meticulous four-step process standardized the 16-h recordings into 12-h of audio data, and sample households’ results of AWC, CTC, and CVC were the average of 2 days of recording. Second, the language ability assessment used in this study was based on self-reported data from the primary caregiver of each child. Therefore, even though the MCDI has been demonstrated to be accurate, reliable, and valid in China (Fenson et al., 2007; Tardif et al., 2008; Zhang et al., 2015; Ma et al., 2021), self-reported data also introduces the potential of reporter bias. Next, the results in this study are correlational, not causal, thus must be interpreted without causality. Moreover, due to few statistical analyses being conducted, we must be cautious to not over-interpret the strength of the significant correlations found. Although we identify correlations between the home language environment and language abilities, future studies should apply experimental or longitudinal research designs to identify the factors that causally influence the early childhood development in rural households in China.

## Conclusion

Evidence from this study shows that within a community of similarly disadvantaged, low-SES households in rural China, a strong association between the home language environment and early language ability exists. Additionally, there was large and substantial variation in measures of the home language environment in rural households. These findings suggest the greater need for interventions targeting improvement of the home language environment for better ECD outcomes, as well as a push for more research on rural communities across China. Indeed, the study shows that there is a significant, pressing need to enhance the policies and programs that would support early childhood development for families in low-SES communities in China. Children’s early language delays can have significant long-term, negative impacts on their future performances, as early childhood development plays a critical role in human capital development. Using LENA technology to further study how the home language environment and early childhood development are related in other low-SES communities in other regions, practitioners and policymakers can develop more targeted interventions to improve children’s language development.

## Data availability statement

The raw data supporting the conclusions of this article will be made available by the authors, without undue reservation.

## Ethics statement

This study received ethical approval from the Stanford University Institutional Review Board (Protocol ID 49552). Trained members of the field survey team received informed oral consent from all caregivers of sample children. Caregivers were aware that their audio recording data would be collected and used for the purposes of this study.

## Author contributions

SR, YM, XZ, TF, and S-ED designed the study and contributed to editing of the manuscript. XZ and YM were responsible for data collection. XZ, YM, and LP analyzed and interpreted the data. LP, XZ, VZ, XW, ML, QL, and ZT drafted the initial manuscript. All authors contributed to the article and approved the submitted version.

## Funding

This study was supported by private gifts from individual donors. The funders had no role in study design, data collection and analysis, decision to publish, or preparation of the manuscript.

## Conflict of interest

The authors declare that the research was conducted in the absence of any commercial or financial relationships that could be construed as a potential conflict of interest.

## Publisher’s note

All claims expressed in this article are solely those of the authors and do not necessarily represent those of their affiliated organizations, or those of the publisher, the editors and the reviewers. Any product that may be evaluated in this article, or claim that may be made by its manufacturer, is not guaranteed or endorsed by the publisher.
