# Supplementary material for: The home language environment and early language ability in rural Southwestern China
Source: Front Psychol. 2023 Mar 17;13:1010442. doi: 10.3389/fpsyg.2022.1010442 (PMC10064000; doi:10.3389/fpsyg.2022.1010442)
Supplement: Supplementary file 1 [file Data_Sheet_1.docx]

Supplementary Material

# Supplementary Figures and Tables

Appendix 1. Differences in demographic characteristics between LENA consent and reject households

| Variables | Gave LENA recording consent | Did not give LENA recording consent | Difference |
| --- | --- | --- | --- |
|  | (1) | (2) | (3) = (2) - (1) |
| **Child characteristics** |  |  |  |
| Age (months) | 21.880 | 22.240 | 0.359 |
|  | [1.603] | [1.967] | (0.361) |
| Gender (1 = boy) | 0.597 | 0.531 | -0.066 |
|  | [0.494] | [0.507] | (0.105) |
| Prematurity (1 = yes) | 0.130 | 0.063 | -0.067 |
|  | [0.338] | [-0.246] | (0.066) |
| **Household characteristics** |  |  |  |
| Age of mother (years) | 27.519 | 28.469 | 0.949 |
|  | [4.248] | [5.236] | (0.958) |
| Maternal education (1 = completed high school or above) | 0.506 | 0.375 | -0.131 |
|  | [0.503] | [0.492] | (0.105) |
| Mother has a job (1 = yes) | 0.610 | 0.500 | -0.110 |
|  | [0.491] | [0.508] | (0.104) |
| Mother is the primary caregiver (1 = yes) | 0.481 | 0.594 | 0.113 |
|  | [0.503] | [0.499] | (0.106) |
| Paternal education (1 = completed high school or above) | 0.468 | 0.290 | -0.177 |
|  | [0.502] | [0.461] | (0.104) |
| Father lived at home for at least 6 months of the past year (1 = yes) | 0.312 | 0.281 | -0.030 |
|  | [0.466] | [0.457] | (0.097) |
| Number of adults in the household | 2.429 | 2.563 | 0.134 |
|  | [1.081] | [1.343] | (0.245) |
| Asset index (PCA score) | 0.101 | -0.244 | -0.346 |
|  | [1.293] | [1.646] | (0.295) |
| Source: authors’ survey. | | | |
| Standard deviations in brackets. Standard errors in parentheses ** p < 0.01, * p < 0.05 |  |  |  |

Appendix 2. Summary statistics and percentile distributions of LENA-generated initiator of Conversation Turns in audio segments and initiator of audio segments

| LENA measures/percentile | Mean/SD | 5th | 25th | 50th | 75th | 95th |
| --- | --- | --- | --- | --- | --- | --- |
|  | (1) | (2) | (3) | (4) | (5) | (6) |
|  |  |  |  |  |  |  |
| Child-initiated CT | 313 | 76 | 193 | 281 | 407 | 599 |
|  | (158) |  |  |  |  |  |
| Female-initiated CT | 270 | 66 | 181 | 246 | 333 | 485 |
|  | (136) |  |  |  |  |  |
| Male-initiated CT | 59 | 7 | 19 | 38 | 79 | 194 |
|  | (57) |  |  |  |  |  |
| Child-initiated segment | 432 | 189 | 331 | 436 | 515 | 733 |
|  | (149) |  |  |  |  |  |
| Female-initiated segment | 380 | 182 | 321 | 387 | 448 | 588 |
|  | (105) |  |  |  |  |  |
| Male-initiated segment | 108 | 18 | 43 | 96 | 152 | 262 |
|  | (81) |  |  |  |  |  |
| Source: Author's survey.  Notes: CT = Conversational Turn; SD = Standard Deviation. | | | | | | |

Appendix 3. Correlations between home language environment measurements, demographic characteristics, and child language development (holding control variables constant)

| Variables | **MacArthur-Bates Communicative Development Inventories (MCDI)** | | |
| --- | --- | --- | --- |
|  | (1) | (2) | (3) |
| **Home language environment measurements** |  |  |  |
| Adult Word Count | 0.001 |  |  |
|  | (0.001) |  |  |
| Conversational Turn Count |  | 0.024* |  |
|  |  | (0.011) |  |
| Child Vocalization Count |  |  | 0.008* |
|  |  |  | (0.004) |
| **Child characteristics** |  |  |  |
| Age (months) | 6.982** | 6.472** | 6.122** |
|  | (1.874) | (1.894) | (1.926) |
| Gender (1 = boy) | -7.025 | -7.490 | -7.775 |
|  | (6.380) | (6.303) | (6.290) |
| Prematurity (1 = yes) | -15.748 | -12.203 | -12.845 |
|  | (8.742) | (8.793) | (8.699) |
| **Household characteristics** |  |  |  |
| Age of mother (years) | -0.224 | -0.214 | -0.172 |
|  | (0.746) | (0.737) | (0.733) |
| Maternal education (1 = completed high school or above) | 1.583 | 1.722 | 1.159 |
|  | (6.358) | (6.283) | (6.244) |
| Mother has a job (1 = yes) | -10.715 | -9.012 | -7.248 |
|  | (10.694) | (10.336) | (10.210) |
| Mother is the primary caregiver (1 = yes) | -11.615 | -8.309 | -7.107 |
|  | (10.583) | (10.317) | (10.289) |
| Paternal education (1 = completed high school or above) | 4.494 | 3.681 | 5.042 |
|  | (6.082) | (6.037) | (5.853) |
| Father lived at home for at least 6 months of the past year (1 = yes) | -6.055 | -6.674 | -5.999 |
|  | (7.529) | (7.455) | (7.408) |
| Number of adults in the household | 4.674 | 4.971 | 3.708 |
|  | (3.180) | (3.152) | (3.107) |
| Asset index (PCA score) | 5.973* | 5.311* | 5.571* |
|  | (2.452) | (2.398) | (2.390) |
| Observations | 77 | 77 | 77 |
| R-squared | 0.361 | 0.375 | 0.380 |
| Source: authors’ survey. | | | |
| Standard errors in parentheses; ** p < 0.01, * p < 0.05 |  |  |  |
